# Microwave Speech Recognizer Empowered by a Programmable Metasurface

**DOI:** 10.1002/advs.202309826

**Published:** 2024-02-21

**Authors:** Hongrui Zhang, Hengxin Ruan, Hanting Zhao, Zhuo Wang, Shengguo Hu, Tie Jun Cui, Philipp del Hougne, Lianlin Li

**Affiliations:** ^1^ State Key Laboratory of Advanced Optical Communication Systems and Networks School of Electronics Peking University Beijing 100871 China; ^2^ Peng Cheng Laboratory Shenzhen Guangdong 518000 China; ^3^ State Key Laboratory of Millimeter Waves Southeast University Nanjing 210096 China; ^4^ Pazhou Laboratory (Huangpu) Guangzhou Guangdong 510555 China; ^5^ Univ Rennes CNRS, IETR—UMR 6164 Rennes F‐35000 France

**Keywords:** artificial intelligence (AI), human–machine interactions, microwave sensing, programmable metasurface, speech recognition

## Abstract

Speech recognition becomes increasingly important in the modern society, especially for human–machine interactions, but its deployment is still severely thwarted by the struggle of machines to recognize voiced commands in challenging real‐life settings: oftentimes, ambient noise drowns the acoustic sound signals, and walls, face masks or other obstacles hide the mouth motion from optical sensors. To address these formidable challenges, an experimental prototype of a microwave speech recognizer empowered by programmable metasurface is presented here that can remotely recognize human voice commands and speaker identities even in noisy environments and if the speaker's mouth is hidden behind a wall or face mask. The programmable metasurface is the pivotal hardware ingredient of the system because its large aperture and huge number of degrees of freedom allows the system to perform a complex sequence of sensing tasks, orchestrated by artificial‐intelligence tools. Relying solely on microwave data, the system avoids visual privacy infringements. The developed microwave speech recognizer can enable privacy‐respecting voice‐commanded human–machine interactions is experimentally demonstrated in many important but to‐date inaccessible application scenarios. The presented strategy will unlock new possibilities and have expectations for future smart homes, ambient‐assisted health monitoring, as well as intelligent surveillance and security.

## Graphical abstract

This work presents an experimental prototype of a microwave speech recognizer empowered by programmable metasurface that can remotely recognize human voice commands and speaker identities even in noisy environments and in the presence of masks or obstacles. Based on voice‐modulated microwave biosignals, this system can enable privacy‐respecting voice‐commanded human–machine interactions in many important application scenarios.

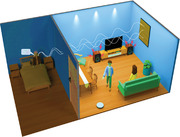

## 1 Introduction

Voice commands are arguably the most natural approach to human–machine interfaces because speech is the most direct communication method between humans. However, the most obvious approach for the machine to capture voice commands, namely the acquisition of the acoustic signals that are the primary information carrier, precludes many important deployment scenarios. On the one hand, voice commands can be drowned in ambient noise under operation in noisy environments such as streets, public transport, or restaurants. On the other hand, it is impossible to operate under silent‐speech requirements^[^ ^1^ ^]^ which may arise to preserve privacy, for use in quiet settings like libraries, or through verbally impaired users (e.g., post‐laryngectomy). Therefore, a wide variety of indirect secondary carriers of information about voice commands have been explored. Many of these techniques achieve high accuracy at the price of being highly invasive because they rely on placing sensors (e.g., magnetic,^[^ ^2^ ^]^ surface electromyographic,^[^ ^3^ ^]^ infrared,^[^ ^4^ ^]^ electropalatographic,^[^ ^5^ ^]^ electromagnetic^[^ ^6^, ^7^ ^]^) directly on the human's body to detect subtle vibrations that are correlated with the speech production. Obviously, such contact‐based approaches are oftentimes inconvenient and, moreover, incompatible with large‐scale deployment in our daily lives. Similar limitations apply to contactless radar‐like approaches based on the emission and reception of acoustic^[^ ^7^, ^8^, ^9^ ^]^ or electromagnetic^[^ ^10^, ^11^ ^]^ waves in the very close proximity (a few centimeters) of the speaker's face. A popular contactless technique for speech recognition that can operate remotely uses optical image sequences as secondary information carrier to recognize speech by analyzing lip or face motion.^[^ ^12^, ^13^ ^]^ However, such visual speech‐recognition methods fail under unfavorable lighting conditions such as darkness as well as when the line of sight from the camera to the speaker's mouth is obstructed by a wall or, more recently, a face mask. In addition, the acquisition of camera images risks to infringe the user's privacy. Bi‐modal speech recognition approaches, combining, for instance, acoustic and visual inputs,^[^ ^14^ ^]^ benefit from richer input information but are also unable to tackle, for instance, the recognition of speech uttered behind a face mask in a noisy environment. An ideal voice‐commanded human‐machine interface would remotely capture relevant biosignals in a robust, noise‐resilient, and privacy‐respecting manner while being cheap, consuming little power, and being easy to deploy in our daily lives, even when the speaker's mouth is hidden behind an optically opaque tissue or wall.

The use of microwaves as remote contactless secondary information carrier of voice commands is predestined to meet this formidable challenge. The ability of microwaves as remote, non‐ionizing sensing technology to penetrate through visually opaque layers is well known, for example, from airport security checks.^[^ ^15^ ^]^ However, capturing microwave biosignals that bear sufficient information about the sought‐after voice commands is itself very challenging because most signal variations may be due to motion that is not related to speech. Therefore, it is of pivotal importance to focus the microwaves on the speaker's mouth, which in turn requires real‐time tracking of the mouth as a pre‐requisite. By focusing on the mouth, the weight of reflections from the region of interest (ROI) in the measured signals is drastically increased. Notable results toward that goal were reported^[^ ^16^ ^]^ through a multiple‐input‐multiple‐output (MIMO) beamforming approach at 2.4 GHz‐WiFi frequencies. We also note that similar impressive radar‐based results have been recently reported in microwave frequencies.^[^ ^7^ ^]^ However, the underlying multi‐channel coherent emission is costly and cumbersome because it requires synchronized sources and individual IQ modulation on each channel. Moreover, using only a few antennas, the setup's degrees of freedom were quite limited, resulting in, for instance, a focal spot that was so large that even winks perturbed the measured signals. A large antenna array emitting coherently controlled wavefronts would be necessary to efficiently localize and focus on the speaker's mouth. Yet this hardware is too costly and power hungry for widespread deployment in human–machine interaction.

**Figure 1:**
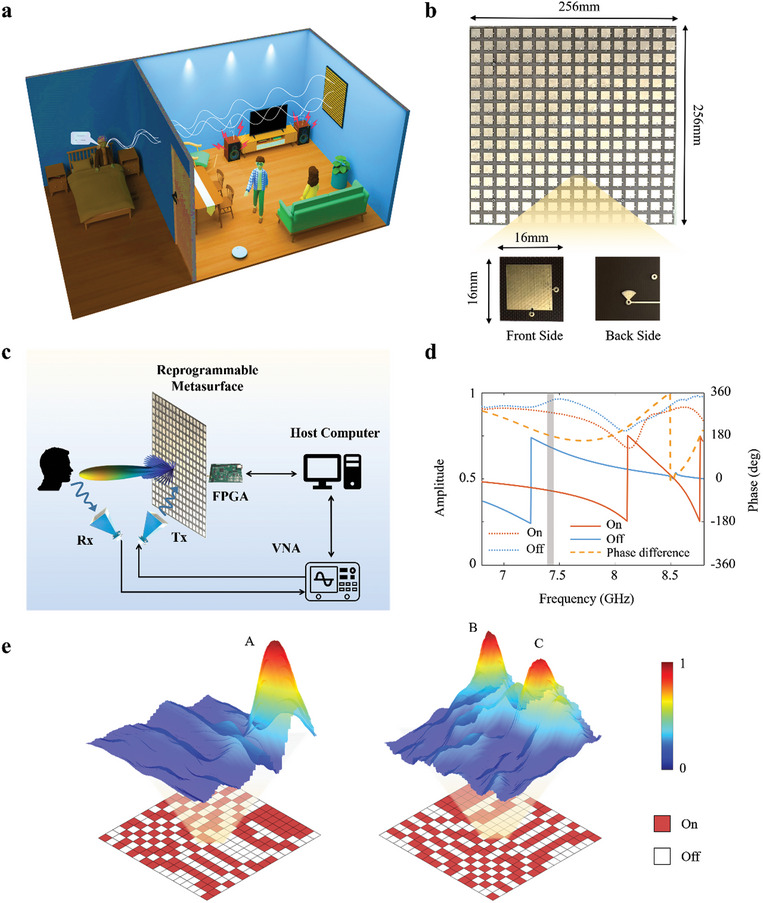
System design of the metasurface‐empowered microwave speech recognizer. a) Conceptual illustration of the proposed microwave speech recognizer in a challenging indoor scenario: an elderly person in the sleeping room voice‐commands an appliance (e.g., lights) through a wall and despite loud music and motion in the neighboring guest room. b) Photographic image of one out of four panels of our one‐bit programmable coding metasurface. The insets show the front and back sides of the designed individual meta‐atom. c) Schematic drawing of the hardware configuration of our proposed microwave speech recognizer, where the volunteer faces directly the metasurface and their body is aligned parallel to the normal direction of the metasurface. The transmitter and receiver are placed on the left and right sides as seen in the schematic, respectively; the transmitter faces the center of the programmable metasurface, and the receiver faces the subject's lips. The hardware of our system consists of a large‐aperture one‐bit reprogrammable metasurface, a pair of horn antennas, a vector network analyzer (VNA) and a host computer. d) Simulated characterization of the frequency‐dependent phase and amplitude response of our designed meta‐atoms in their two possible configurations (“0”/‘ON” and “1”/‘OFF”). e) Maps of the spatial distribution of the microwave field magnitude after normalization (measured via near‐field scans) corresponding to the indicated metasurface configurations that are chosen to focus on point A (left) or points B and C (right).

In this article, we show that a deep‐learning‐controlled programmable metasurface fully reaps the benefits of microwave speech recognition with a drastically simpler hardware. Our programmable metasurface^[^ ^17^ ^]^ is an array of 1024 meta‐atoms with individually controllable reflection properties, fed by a single source. Compared to a conventional antenna array comprising a few antennas, we have thus three orders of magnitude more degrees of freedom and, moreover, a much larger aperture. Building on recent results from intelligent computational meta‐imaging,^[^ ^18^ ^]^ these advantages allow us to localize and focus on the speaker's mouth with very high efficiency. We use this ability to prototype a voice‐commanded human–machine interaction scenario in which a speaker who is hidden behind a wall commands a mechanical hand. Our system is capable of tracking a moving speaker in real time, dynamically generating suitable spatial beams for focusing on the speaker's mouth and interpreting the measured biosignals with a deep‐learning technique. We also demonstrate multi‐speaker listening. Moreover, we shed new light on the mechanisms through which speech information is encoded in microwave biosignals: we demonstrate that, besides the obvious reflection off the mouth, the probing microwave signals partially penetrate through the skin and are affected by the tongue and other vocal entities. Finally, we evidence that our system can also be utilized as biometric identification technology because of the individual manner in which each subject utters speech. Our experimental results enable voice‐commanded human–machine interaction at minimal cost in a plethora of challenging and to‐date inaccessible scenarios such as health care for assisted living (see **Figure** **1**a for an example); our results may also be valuable in security applications requiring intelligent surveillance.

## 2 Results and Discussion

### 2.1 System Configuration

We start by elaborating on the system configuration of our implemented prototype for our proposed metasurface‐empowered microwave speech recognition. On the hardware level, our system comprises a large‐aperture one‐bit programmable metasurface (1024 meta‐atoms, 51.2 cm × 51.2 cm aperture), a pair of commercial horn antennas, a vector network analyzer (VNA, Agilent E5071C), and a host computer—see Figure 1c. Our programmable‐metasurface‐empowered system must accomplish a series of complex sensing tasks on the fly. First, our system must localize the speaker's mouth. This involves imaging the scene and interpreting the resulting image to identify the ROI, that is, the mouth. Second, our system must focus microwaves on the mouth and capture the reflected biosignals. The focusing is important to efficiently suppress the influence of unwanted clutter originating from other body parts and the surrounding environment, enabling a high signal‐to‐clutter ratio (SCR). Third, our system must interpret the measured biosignals to extract the sought‐after speech content.

Our system's autonomous ROI identification and analysis^[^ ^19^ ^]^: during the first step, the scene is imaged with a series of 18 random illuminations, generated through a known series of 18 random metasurface configurations. The measured data is processed by artificial intelligence (AI) tools to generate a 3D skeleton of the speaker. Details of the utilized AI architecture are provided in Note S1. (Supporting Information) Based on the estimated skeleton, the coordinates of the mouth, our ROI, can be deduced. Using a modified Gerchberg–Saxton algorithm, our system then identifies a metasurface configuration that focuses microwaves on the speaker's mouth. A series of probing focused microwave signals is emitted with a period of 70 ms, and the reflected signals are captured by the receiving horn antenna. Note that the use of directive horn antennas, in contrast to omnidirectional antennas, further helps with discriminating ROI signals from multipath scattered signals in the room. The measured biosignals are then interpreted by an artificial neural network (ANN) that directly maps the acquired microwave data to the desired speech content. Such a direct transcription of measured signals with text, without intermediate steps involving phonetic representations, is known as “end‐to‐end” speech recognition in the signal‐processing literature.^[^ ^20^ ^]^ Inspired by ref. [21], we have developed a customized microwave‐speech transformer for our system and trained it with supervised learning. To obtain the labeled microwave‐speech training data, we used the host computer's built‐in microphone that was synchronized with our proposed microwave speech recognizer. Algorithmic details about our microwave‐speech transformer are provided in Note S1 (Supporting Information).

The first step of our system's pipeline is a conventional instance of compressive imaging by leveraging the configurational diversity of a programmable meta‐imager, first reported^[^ ^22^ ^]^ (see also Sec. II.B in ref. [18] for a balanced review of the field). Nonetheless, our system's pipeline in its entirety qualifies as an instance of “intelligent meta‐imaging” according to the taxonomy^[^ ^18^ ^]^ (see Sec. III.A therein) because AI tools influence the choice of task‐specific hardware configurations for our metasurface in the second step of its complex sequences of tasks. We note that related techniques for autonomous ROI identification were put forward in optical ghost imaging based on analytical rather than AI tools.^[^ ^23^, ^24^, ^25^ ^]^ However, our AI‐driven sensing pipeline is distinct from another class of intelligent meta‐imagers which integrates the programmable meta‐atoms as trainable physical weights directly into an end‐to‐end pipeline comprising both the physical and digital layers^[^ ^26^, ^27^ ^]^ (see Sec. III.B‐C in ref. [18]). The latter could be a future extension for the first step of our scheme.

The pivotal hardware ingredient of our microwave speech recognizer is an inexpensive one‐bit programmable coding metasurface composed of 32 × 32 electronically controllable meta‐atoms. A photographic image of a 16 × 16 panel of meta‐atoms is shown in Figure 1b. Each meta‐atom has dimensions of 16 mm × 16 mm × 1.88 mm and consists of a five‐layer structure. A PIN diode (MADP‐000907‐14020x) is embedded on the top layer. By controlling the bias voltage of the PIN diode, we can electronically switch between two distinct meta‐atom reflection properties in the microwave domain: The 0°‐phase (denoted as digit ‘0′) and 180°‐phase (denoted as ‘1′) states are achieved when the PIN diode is biased with an externally applied DC voltage of 5 V (ON) or 0 V (OFF), respectively. The frequency‐dependent magnitude and phase responses of our designed meta‐atom are simulated using the commercial software package CST Microwave Studio 2000, and the results are plotted in Figure 1d. Our designed meta‐atom can efficiently manipulate the reflected electromagnetic field within the frequency range from 7.49 to 8.3 GHz: the reflection phase of the meta‐atom shows a roughly 180° phase difference when the embedded PIN diode is switched from the ON state to the OFF state, while the reflection magnitudes are almost the same and close to unity. Thus, the radiation pattern can be flexibly manipulated by suitably biasing the PIN diodes of the metasurface through a field‐programmable gate array (FPGA). More details about the programmable metasurface are provided in Note S2 (Supporting Information).

To examine the crucial role of our large‐aperture programmable metasurface in discriminating between reflections from the mouth and undesired clutter as well as to improve the SCR, we have conducted a series of preliminary experiments which seek to focus the microwave field at one or two desired location(s). The spatial distributions of the microwave field are obtained through near‐field scans. Two representative experimental results are shown in Figure 1e for focusing on a single point A (0.12 m, 0.14 m, 1 m) or simultaneously on two points B (−0.05 m, 0.14 m, 0.65 m) and C (0.1 m, 0.14 m, 0.65 m), respectively, which were experimentally obtained via so‐called near‐field scanning technique.^[^ ^28^, ^29^ ^]^ The corresponding metasurface configurations are identified via a modified Gerchberg–Saxton algorithm and also displayed in Figure 1e. These results demonstrate the ability of our system to reallocate the microwave energy to one or multiple desired spot(s) in a dynamic manner using a suitable configuration of our programmable metasurface. The signal level at the desired spots is enhanced by a factor of around 20 dB. This focusing on the ROI is crucial in order to drastically increase the weight of reflections from the ROI and to suppress the influence of undesired clutter, noise and multipath reflections.

### 2.2 Encoding of Speech Information in Microwave Biosignals

**Figure 2:**
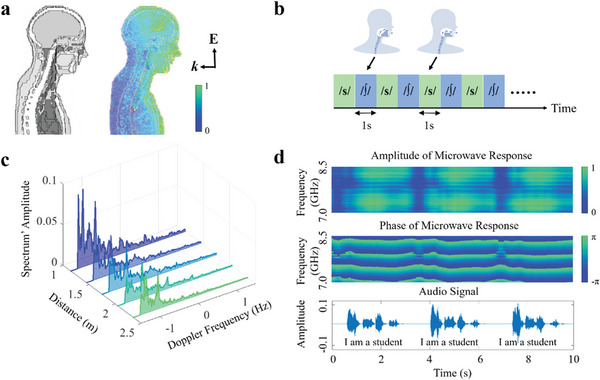
Physical mechanisms of speech encoding in the proposed microwave speech recognizer. a) Full‐wave simulation of the interaction of the chosen microwave signal with the vocal organs, which is achieved by using a full‐wave simulator, CST Microwave Studio 2012. In our simulation, the subject (Laura, a model of a 43‐year‐old female from ref. [30]) is illuminated with a linearly polarized plane wave. b) The experimental scheme according to which the subject is asked to alternately pronounce an alveolar |s| and a cacuminal |∫|, with a period of two second for a duration of 5 min. c) The experimental results corresponding to the scheme presented in b: the amplitudes of the microwave biosignals are plotted as a function of the Doppler frequency. These measurements are taken at five different distances between the subject and the programmable metasurface (0.9 m, 1.2 m, 1.5 m, 1.8 m, and 2 m). d) Selected microwave responses when the subject is asked to pronounce a short sentence, i.e., ‘I am a student’, three times. In addition, the corresponding sound signal acquired by the in‐built microphone of our host computer is plotted for comparison.

Having described our system configuration, we now wish to investigate the mechanisms through which speech information is encoded in the microwave biosignals that our system acquires. The acoustic sounds that constitute a voice originate from air being exhaled by the lungs and causing vocal cord vibrations. The sound is additionally shaped through a series of further articulatory entities such as tongue position and mouth opening. Clearly, the more the acquired signals of a remote sensing technique interact with all of the involved articulatory entities, the more speech information is expected to be encoded in the biosignals. Contactless visual speech recognition techniques solely rely on mouth motion because optical frequencies cannot penetrate through the skin and teeth. Similarly,^[^ ^7^, ^16^ ^]^ based on a remote contactless microwave approach suggests it relies solely on mouth motion. In contrast, ref. [6] reported that interior articulatory entities like the tongue decisively impact their contact‐based measurements in the microwave regime. In fact, ref. [6] observed less microwave interaction with interior articulatory entities when the speaker had metallic tooth fillings, a clear indicator that the microwaves penetrated through the skin. These findings^[^ ^6^ ^]^ are in line with our observations. As displayed in **Figure** **2**a, for our working frequency around 8 GHz, we find in full‐wave simulations (CST Microwave Studio) that a significant portion of the microwave signal penetrates through the skin and teeth and interacts notably with the subject's articulatory entities such as the tongue. In addition, the spatial resolution is decent because the wavelength at 8 GHz is 3.75 cm in free space and even less inside the biological tissue. These results suggest that our technique is remarkably different from existing non‐microwave‐based strategies in that it can efficiently encode speech information not only from the mouth but also from interior articulatory entities such as the tongue.

To test this hypothesis experimentally, we next conduct a series of speech experiments with our microwave speech recognizer system. Therein, the subject pronounces alternately two syllables, an alveolar |s| and a cacuminal |∫|, and repeats them with a period of 2 sec for 5 min (see Figure 2b). The volunteer is asked to keep the mouth as close as possible during the whole pronunciation process so that only tongue motion inside the mouth is involved. Thereby, we minimize the encoding of speech information in the microwave biosignals through the mouth. If our system is nonetheless capable of extracting speech information, this proves that it efficiently probes articulatory entities other than the mouth, too. The amplitudes of the acquired microwave biosignals as a function of the Doppler frequency are plotted in Figure 2c; each curve is averaged over 100 repeated acquisitions, and the experiment was performed for five different distances between the speaker and the metasurface (*d*  =  0.9 m, 1.2 m, 1.5 m, 1.8 m, and 2 m). The corresponding sizes of the focal spot on the mouth are estimated to be on the order of $O(\frac{\lambda d}{D})$, where *D*  =  51.2 cm is the metasurface aperture, yielding values between 6.6 cm and 14.6 cm. As expected, the amplitude responses show the peaks at the Doppler frequency of around 0.5 Hz in all five cases. It is evident that the microwave signals can capture the hidden vocal vibrations and motions even though the motion of the subject's skin (lip and face) cannot be visually perceived. These microwave signals could be further processed to infer the speech content.

As a last set of preliminary experiments, the speaker is asked to pronounce a larger number of syllables and repeat them for 5 min with a frequency of 0.5 Hz per syllable. The corresponding microwave responses are collected by the developed microwave speech recognizer. Figure 2d shows amplitude and phase of the microwave responses corresponding to five different syllables. The corresponding sound signals obtained from the built‐in microphone of the host computer are also plotted for comparison. It can be seen from Figure 2d that the microwave responses are correlated with the corresponding sound signals in the time domain. The frequency spectrum of the microwave responses has distinct properties for each syllable.

### 2.3 Microwave Speech Recognition in Line‐of‐Sight Setting

**Figure 3:**
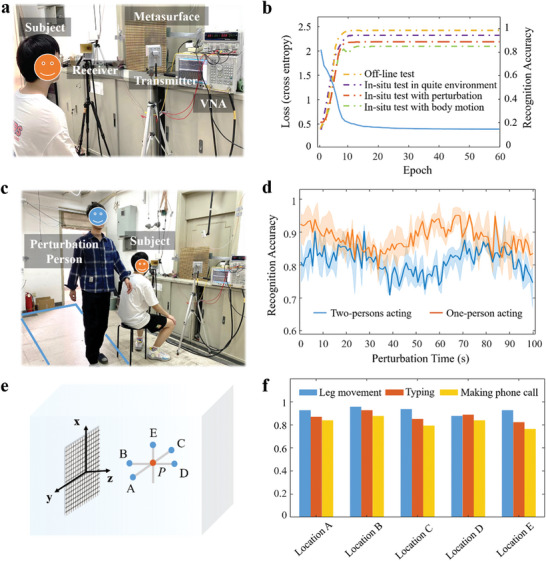
Experimental results of the microwave speech recognition in a line‐of‐sight setting. a) Experimental setting, where the subject is wearing or not wearing a face mask and sitting in front of the reprogrammable metasurface. b) The training and test behaviors of the microwave speech recognizer as a function of the training epoch. The logarithm of the loss function is plotted as blue solid line (left axis), and the test behavior is examined in terms of the recognition accuracy (right axis). Here, we consider four test cases with the microwave speech recognizer trained in the quiet environment: the simple test with the off‐line collected test samples (called off‐line test), the in‐situ test with a subject in the same quiet environment as that for the training (called in‐situ test in quiet environment), the in‐situ test with a subject disturbed by an additional person freely acting in room (called in‐situ test with perturbation), the in‐situ test with a subject with different body motions (called in‐situ test with body motion). c) Experimental setting for the investigation of the microwave metasurface speech recognizer's robustness to the disturbances of the ambient environment: an additional person acts freely within the region marked by the blue box while the subject reads out loud the assigned material. d) Experimental speech recognition results for the scene from "c" under the interference from motions of one or two other people. We make multiple repeated tests and the curve is the average of the accuracy at a certain time. The filled shaded area indicates its fluctuation range. e) Experimental setting for examining the robustness when the speaking subject makes three different kinds of body motions (making phone call, typing, rhythmical leg movement) at five different locations A (0.04 m, 0.1 m, 1.15 m), B (0.04 m, 0 m, 1.0 m), C (0.04 m, −0.1 m, 1.15 m), D (0.04 m, 0 m, 1.3 m), and E (0.08 m, 0 m, 1.15 m), while reading out loud. f) Experimental speech recognition results for the setting from **e**.

Here, we begin to examine the performance of the developed microwave speech recognizer in a line‐of‐sight scenario, where a subject sits in front of the metasurface, as seen in **Figure** **3**a. To train our metasurface speech recognizer, we have recruited 22 volunteers (7 20‐to‐25‐year‐old graduate students and 15 17‐year‐old undergraduate students; 5 females and 17 males) and aim at recognizing 100 daily used English words (see Note S3, Supporting Information). In the experiments, all volunteers were asked to read out loud the designated material five times at a normal speed (half a word per second on average) in a quiet electromagnetic environment. By “quiet electromagnetic environment” we mean that there is no disturbance of the acquired microwave signals by motion not related to the speech. As mentioned above, the built‐in microphone in our host computer is utilized for collecting the labeled voice data for supervised learning. In this way, we have acquired a total of 11 000 pairs of labeled microwave‐voice samples per location, in which 70% of the samples are randomly selected for training the microwave‐voice transformer, and the rest are used for testing. Selected samples are provided in Note S4 (Supporting Information)

First, we consider the simple case in which a single subject with or without wearing a surgical face mask sits at *P* (0.04 m, 0 m, 1.15 m) in front of the reprogrammable metasurface in a static and quiet environment, as shown in Figure 3a. Figure 3b demonstrates the learning and test performances of the developed microwave speech recognizer in terms of how the loss function evolves over the course of the training epochs. In addition, the dependence of the speech recognition accuracy over the training samples as the epoch index increases is plotted in Figure 3b. We mix cases in which the subject wears a face mask or not because the results are almost identical in both cases. Figure 3b shows that the developed speech recognizer can be effectively trained to achieve near‐perfect speech recognition, and that the trained system works very well on the “unseen” test samples, even when the speaker wears a mask. In other words, these results indicate that the proposed microwave speech recognizer can “hear” what people say without audio and visual clues and “see” the speaker's mouth in a remote‐sensing manner.

Next, we evaluate the robustness of our speech recognition procedure in a dynamically changing environment. Body motion or a changing environment can lead to parasitic variations of the acquired microwave signals that could deteriorate the speech recognition performance. We conducted a set of experiments in which a second person, referred to as the “perturbation person,” acts freely within the indicated region in Figure 3c while the subject reads out loud the designated material. To interpret the microwave signals we use the artificial neural network previously trained in the aforementioned quiet environment. The results in this dynamically changing environment are shown in Figure 3d and demonstrate the robustness of our approach. This robustness can be attributed to the efficiency with which our programmable metasurface focuses the microwave beam on the subject's mouth such that reflections off the perturbation person are very weak and hence their disturbing impact is efficiently suppressed.

We also examined the robustness with respect to body motion of the speaking subject. Here, we consider a realistic scenario in which the subject walks around freely while speaking. This implies that the distance and orientation of the subject's mouth relative to the programmable metasurface are varying. Specifically, the subject spoke at the five different locations around the point *P*, indicated in Figure 3e while performing three different kinds of body motion (making a phone call, typing, rhythmical leg movements). The achieved recognition accuracies, based on the microwave‐voice transformer network trained in the quiet environment, are reported in Figure 3f. These results indicate that the microwave speech recognition performance is almost unchanged when the subject makes phone calls or types while reading the designated text. Again, this robustness can be attributed to our efficient programmable‐metasurface system that tracks the subject's mouth and ensures that the microwave focal spot follows the subject's motion. These characteristics are very encouraging with regard to real‐life convenient and robust speech sensing, irrespective of the speaker's distance and orientation.

### 2.4 Microwave Speech Recognition in Through‐a‐Wall Setting

Now, we consider a more challenging setting, i.e., microwave multi‐speaker speech recognition in a through‐a‐wall scenario. To that end, we asked two subjects to sit behind a 5 cm‐thick wooden wall and talk to each other. The wall has a higher dielectric constant than air and possibly an additional microstructure, which previously motivated the conception of special wall‐compensation algorithms to mitigate artefacts due to reflections and wavefront distortions in through‐a‐wall computational meta‐imaging.^[^ ^31^ ^]^ The multi‐speaker problem now requires that the metasurface is configured such that it simultaneously focuses microwave energy on both speakers’ mouths‐like the example from Figure 1e. To train the microwave metasurface speech recognizer, 22 volunteers read the assigned English reading material five times behind the 5 cm‐thick wooden wall. As before, 70% of the samples are randomly selected for training the microwave‐voice transformer, and the rest are used for testing. The corresponding experimental results have been reported in Note S5 (Supporting Information). The recognition accuracies of above 80% from Figure S4 (Supporting Information) confirm that our system performs well also for the very challenging multi‐speaker through‐a‐wall speech recognition task, even if a third person acts freely in the room while the two subjects talk to each other. We believe that the above two‐speaker results can be extended to settings involving more speakers, and extensions to dealing with the so‐called “cocktail party problem” may be possible.

### 2.5 Voice‐Commanded Human–Machine Interaction

Besides recognizing the content of voice commands which is the problem we have studied so far, ideally, a voice‐commanded human‐machine interface should additionally be able to recognize the speaker's identity. Voice is well‐established as acoustic “fingerprint” of a user's identity because unique vocal features are encoded by unique properties of the individual's lung, vocal cords, vocal tract, and other articulatory entities. Therefore, even though multiple people pronounce the same words, the uttered sounds include distinctive features for each person. We now explore whether user identification is also possible with our acquired microwave biosignals. Interestingly, this variation across different users can be interpreted as a form of noise with respect to speech recognition whereas it is the salient feature for user identification. This double‐sided‐sword nature of signal variations as being either noise or the crucial feature is reminiscent of microwave‐based complex localization problems.^[^ ^32^ ^]^

**Figure 4:**
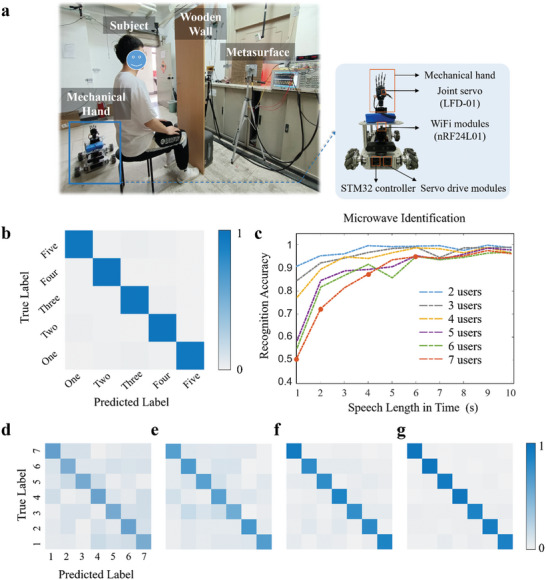
Voice‐commanded through‐a‐wall human‐machine interaction based on our metasurface‐empowered microwave speech recognizer. a) Experimental setting. Details about the mechanical hand are provided in the inserted figure. Further details can be found in Video S1 (Supporting Information). b) Classification confusion matrix for the five different speech commands: “one”, “two”, “three”, “four”, and “five”. c) Results illustrating the dependence of speaker recognition accuracy on the speech length for varying numbers of speakers. d‐g) Classification confusion matrices depicting identification accuracy corresponding sequentially to the four red points marked in "**c"** from left to right.

We consider the through‐a‐wall microwave speech recognition of seven subjects (the aforementioned seven graduate students). Samples are now labelled with classes from 1 to 7 according to which subject they correspond to. The data processing problem is now a multivariate classification problem for which we have developed a deep convolutional neural network (see Note S1, Supporting Information for more details). We explore two factors that we expect to have a major influence on the identification performance: the speech sample length and the number of subjects to be distinguished. Indeed, the results plotted in **Figure** **4**c–g demonstrate that as more subjects must be distinguished, acceptable classification accuracy can be achieved by analyzing longer speech samples. Using only 6 sec‐long speech samples, we can distinguish between all seven individuals based on the microwave biosignals, without any vocal and visual clues.

Finally, we now discuss our demonstration of a voice‐commanded human‐machine interface in which a mechanical hand is controlled based on through‐a‐wall vocal speech that is recognized by our microwave speech recognizer. The corresponding experimental setup is depicted in Figure 4a. The system now recognizes the speech content and subsequently sends out the recognized speech commands to a mechanical hand in order to control the motion of the latter in real time. The mechanical hand is integrated into a mobile vehicle and each finger is fitted with an anti‐blocking joint servo (LFD‐01) for finger retraction control. An on‐board Wi‐Fi module (nRF24L01) is used to wirelessly receive control commands from the host computer based on the recognized vocal commands. The vehicle is equipped with a STM32 controller to process the received control commands into the control quantities for the corresponding finger servos (see Note S6, Supporting Information for details). Five different speech commands are involved in this experiment: “one,” “two,” “three,” “four,” and “five.” We have evenly collected 1000 pairs of microwave and acoustic samples for the five commands and utilized 70% and 30% of samples to train and test the microwave speech recognizer, respectively. We report the classification confusion matrix in Figure 4b, showing that near‐perfect speech recognition of the five commands can be achieved by using our microwave speech recognizer. More details have been recorded in Video S1 (Supporting Information). These experimental results demonstrate the important potential of our microwave speech recognizer for microwave‐based contactless voice‐commanded human‐machine interfaces.

### 2.6 Discussion

Before concluding this work, we briefly discuss two important aspects of the privacy‐respecting properties of the proposed microwave speech recognition. First, the presented strategy is based on microwave signals, which are non‐visual and thus present fundamentally lower privacy concerns than visual techniques. For instance, facial information and other visual personal details are never even acquired. Furthermore, the microwave signal is modulated by the programmable metasurface, implying that the echoes have been encrypted by the programmable metasurface on the physical level.^[^ ^33^, ^34^ ^]^ Thereby, an eavesdropper cannot access information about the subject without knowing the coding pattern of the programmable metasurface. Second, the pretrained microwave‐speech transformer works very well for an unseen subject, implying that there is no need for the enrollment of the subject. To see this more clearly, we have conducted a set of experiments and the corresponding results are reported in Note S7 (Supporting Information) Here, we still use the aforementioned 22 volunteers, but take randomly 14 volunteers for training, and the remaining 8 volunteers for testing. The data from these 8 testing volunteers were organized into 4 groups. Each group contained all data of 2 volunteers. The set of results indicate that, regardless of whether volunteers wear masks or not, the model is able to recognize unseen subjects’ speech.

## 3 Conclusion

To summarize, we have proposed and experimentally prototyped the concept of a microwave speech recognizer empowered by a programmable metasurface, including a demonstration of a voice‐commanded human‐machine interface. Our system answers two fundamental questions in speech recognition—“what is being said?” and “who is speaking?”—based on voice‐modulated microwave biosignals. The unique advantages of a large‐aperture programmable metasurface enable us to implement microwave‐based speech recognition with unprecedented accuracy because we can dynamically track the speaker's mouth and focus microwaves on it with high efficiency. Our work is particularly timely in the current pandemic context: people always wear masks in public places such that their lip movements cannot be seen, and neither can their voice be heard given loud ambient noise sources. The demonstrated ability to implement contactless voice‐commanded human‐machine interfaces without reliance on optical or acoustic cues will enable numerous important but to‐date inaccessible applications of human‐machine interfaces such as in smart health care or industrial settings, as well as intelligent surveillance and security.

## 4 Experimental Section

### Design of the One‐Bit Reprogrammable Coding Metasurface

The reprogrammable coding metasurface is an ultrathin planar array of meta‐atoms that are individually reconfigurable via electronic commands.^[^ ^17^, ^35^, ^36^ ^]^ Thanks to its unique capabilities to manipulate electromagnetic wavefields in a reprogrammable manner, it has elicited many exciting physical phenomena (e.g., nonreciprocal reflection effects^[^ ^37^ ^]^) and versatile functional devices, including computational imagers,^[^ ^18^, ^19^, ^22^, ^26^, ^27^, ^28^, ^38^, ^39^, ^40^, ^41^, ^42^, ^43^ ^]^ dynamic holography,^[^ ^29^ ^]^ wireless communications,^[^ ^44^, ^45^, ^46^, ^47^, ^48^, ^49^, ^50^, ^51^ ^]^ analog wave‐based computing,^[^ ^52^, ^53^, ^54^ ^]^ and dynamic cloaks.^[^ ^55^ ^]^

We have designed a one‐bit reprogrammable metasurface that is composed of 32 × 32 meta‐atoms. The meta‐atom is a five‐layer structure as shown in Figure 1b and Note S2 (Supporting Information). The top layer is a square copper patch with dimensions of 11 mm × 11 mm, which contains a PIN diode to control the reflection phase of the meta‐atom. The second layer has a thickness of 1.58 mm and is made of Taconic TLX‐8 which has a relative permittivity of 2.55. The fourth layer has a thickness of 0.3 mm and is made of FR‐4 with a dielectric constant of 4.3. The third and fifth layers are ground planes made of copper, and a via hole is introduced on the third layer to isolate the bias voltage coming from the fifth layer. For the sake of easy fabrication, the entire reprogrammable coding metasurface is designed to be composed of 2 × 2 metasurface panels, and each panel consists of 16×16 electronically controllable digital meta‐atoms. One such panel is depicted in Figure 1b. Each metasurface panel is equipped with eight 8‐bit shift registers (SN74LV595APW), and eight PIN diodes are sequentially controlled. The adopted clock rate is 50 MHz, and the ideal switching time of the PIN diodes is 10 µs.

### Algorithmic Overview

The first step of our microwave speech recognizer is to localize the ROI in the scene, i.e., the speaker's mouth. To this end, the scene is illuminated with 18 random patterns generated by a known series of random metasurface configurations. The acquired data is directly mapped to a 3D skeleton of the speaker using a deep ANN (see Note S1, Supporting Information).^[^ ^56^ ^]^ Based on the 3D skeleton, it is then straightforward to localize the speaker's mouth. These ROI coordinates are needed to identify a metasurface configuration that efficiently focuses microwaves on the speaker's mouth. A suitable metasurface configuration for this focusing task is identified with a modified Gerchberg−Saxton algorithm based on the ROI coordinates. This is the basis for capturing clutter‐resiliant microwave biosignals from which speech information can be extracted. The autonomous ROI identification here differs from that in ref. [19] in that the acquired data from the first step is mapped to a 3D skeleton as opposed to a full image.

### Microwave‐Speech Transformer

The microwave‐speech transformer is a deep artificial neural network which directly converts the sequence of microwave signals to the sequence of recognized speech information in an end‐to‐end fashion. The architecture is inspired by ref. [21] and detailed in Note S1 (Supporting Information) The network adopts the typical Transformer structure and uses an encoder–decoder module structure, which is mainly composed of multi‐head attention layer, feed‐forward layer, residual connection and layer normalization. The network training is performed using the Adam optimization method^[^ ^57^ ^]^ with a mini‐batch size of 64, an epoch setting of 50, and a learning rate of 3 × 10^−4^. The complex‐valued weights are initialized randomly with a zero‐mean Gaussian distribution of standard deviation 10^−3^. The training is performed on a workstation with an Intel Xeon E5‐1620v2 central processing unit, NVIDIA GeForce GTX 2080Ti, and 128 GB access memory. The machine learning platform TensorFlow is used to define and train the networks.

### Speaker Identity Recognition

The network for recognizing the speaker's identification from the microwave biosignals is based on a simple CNN structure as detailed in Note S1 (Supporting Information). It consists of convolutional layers, pooling layers, fully connected layers, and Softmax activations. The network maps the acquired biosignals directly to the user identity class.

### Configuration of Proof‐of‐Concept System

The experimental setup, as shown in Figure 1b, consists of a transmitting (TX) horn antenna, a receiving (RX) horn antenna, a large‐aperture reprogrammable metasurface, and a vector network analyzer. The two horn antennas are connected to two ports of the VNA via two 4m‐long 50‐Ω coaxial cables, and the VNA is used to acquire the response data by measuring transmission coefficients (*S*_21_). In addition, an in‐build sound microphone in the host computer has been integrated into our system for acquiring labeled training data. The computer controls the VNA and microphone to acquire the microwave data and voice signal, respectively, using the Python 3.1 software. These two procedures of data acquisition share the same starting time and ending time, but with different sampling intervals, 70 ms for the microwave data from the VNA and $\frac{1}{22050}s$ for the acoustic data from the microphone. Note that the rate of signal sampling could be remarkably improved if a specialized device was utilized for the signal acquisition. Thereby, more advanced functionality of microwave speech recognition could be achieved, for instance, the classification of phonemes in lip language. This will be the subject of future work. Note that the sound signals are solely used to assist in labeling the microwave data with corresponding text to obtain labeled training data, since our primary goal is to infer the speech information from the microwave data. To that end, we cut the acoustic signal corresponding to a specific text by listening and writing down the start and end times. Since the microwave speech data and the acoustic data are acquired at the same start time, we can easily align each sampling interval. Finally, we can label the microwave speech data with the corresponding text. We input these microwave biosignals into the neural network and train the latter so that it outputs the corresponding speech.

### Statistical Analysis

All experiments were performed in three or more replicates. Results were expressed as the mean ± standard deviation (SD). For intergroup comparisons, repeated data were examined based on variance analysis. All numerical simulations of the programmable metasurface are performed using a commercial full‐wave EM simulator, CST Microwave Transient Simulation Package 2017. The network design, training, and computational tasks are all executed using the PyTorch library. We also used MATLAB R2020b software for further signal processing and analysis. More details were provided in Supporting Information.

## Conflict of Interest

The authors declare no conflict of interest.

## Author Contributions

H.Zhang., H.R., H.Zhao contributed equally to this work. L.L. conceived the idea and conducted the theoretical analysis. P.d.H. contributed to the conceptualization of the project. All authors contributed to data analysis and interpretation. L.L. and P.d.H. wrote the manuscript, and all authors read the manuscript.

## Supporting information

Supporting Information

Supplemental Video 1

## Data Availability

The data that support the findings of this study are available from the corresponding author upon reasonable request.
