# Supplementary material for: Microwave Speech Recognizer Empowered by a Programmable Metasurface
Source: Adv Sci (Weinh). 2024 Feb 21;11(17):2309826. doi: 10.1002/advs.202309826 (PMC11077686; doi:10.1002/advs.202309826)
Supplement: Supplementary file 1 — Supporting Information [file ADVS-11-2309826-s002.pdf]

ADVANCED SCIENCE
Open Access

Supporting Information

for *Adv. Sci.*, DOI 10.1002/advs.202309826

Microwave Speech Recognizer Empowered by a Programmable Metasurface

Hongrui Zhang, Hengxin Ruan, Hanting Zhao, Zhuo Wang, Shengguo Hu, Tie Jun Cui, Philipp del Hougne* and Lianlin Li**

Supplementary Notes for

Microwave speech recognizer empowered by a programmable metasurface

Hongrui Zhang[1+], Hengxin Ruan[1,2+], Hanting Zhao[1+], Zhuo Wang[1], Shengguo Hu[1],

Tie Jun Cui[3,4*], Philipp del Hougne[5,*], Lianlin Li[1,4*]

[1]State Key Laboratory of Advanced Optical Communication Systems and Networks, School of Electronics, Peking University, Beijing 100871, China

[2]Peng Cheng Laboratory, 518000, Shenzhen, Guangdong, China

[3]State Key Laboratory of Millimeter Waves, Southeast University, Nanjing 210096, China

[4]Pazhou Laboratory (Huangpu), Guangzhou, Guangdong 510555, China

[5]Univ Rennes, CNRS, IETR - UMR 6164, F-35000 Rennes, France

[+] These authors contributed equally to this work.

[*]Corresponding authors: tjcui@seu.edu.cn; philipp.del-hougne@univ-rennes1.fr; lianlin.li@pku.edu.cn

Supplementary Note 1: Deep artificial neural networks

In this supplementary note, we provide details on the three deep artificial neural networks (ANNs) underlying our microwave speech recognizer:

- The microwave-speech transformer that directly maps microwave biosignals to text.
- The convolutional neural network (CNN) that identifies the speaker based on the microwave biosignals.
- The CNN for reconstructing a 3D skeleton of the subject in order to precisely localize the mouth's coordinates.

●The microwave-speech transformer

Fig. S1a reports the structure of the microwave-speech transformer (MST), which maps the sequence of microwave biosignals directly to the sequence of recognized speech information. Such a direct transcription of measured signals with text, without intermediate steps involving phonetic representations, is known as 'end-to-end' speech recognition in the signal-processing literature. Inspired by Ref.[1], our whole network uses an encoder-decoder module structure and every module is composed of multi-head attention layers and feed-forward layers. In addition, the residual structure and layer normalization are applied to prevent the so-called gradient disappearance and accelerate network training. The main difference from traditional transformer is our inputs here is the microwave biosignals, thus they are not embedded but positional encoded directly before entering the encoder. Other parameters of the MST are set as follows: each feed-forward block has a multilayer perceptron (MLP) hidden layer with width of 2048. The label-smoothing rate and dropout rate are set to be 0.1 and 0.3, respectively. Furthermore, we use the so-called Softmax as the activation function and the number of heads is set as 6.

To train the MST, the Adam optimization method is utilized in the TensorFlow environment. The mini-batch size is 64, the number of epochs is 50, and the learning rate is set to 3×10^{-4}. In addition, the complex-valued weights are initialized with a zero-mean Gaussian distribution of standard deviation 10^{-3}. The training is performed on a

workstation with an Intel Xeon E5-1620v2 central processing unit, NVIDIA GeForce GTX 2080Ti, and 128GB access memory.

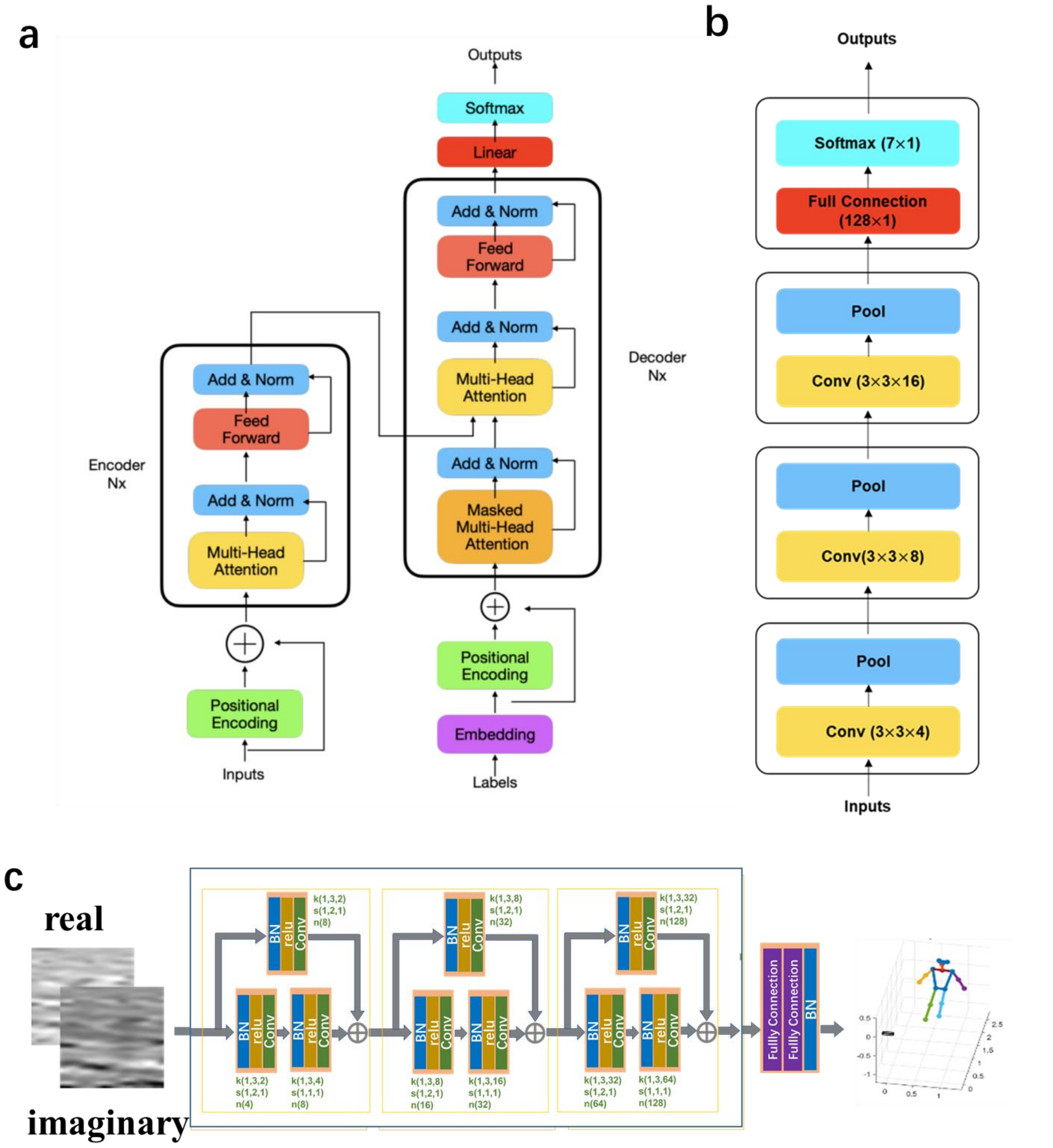

Fig. S1. Architectural details of the three ANNs underlying our metasurface-empowered microwave speech recognizer. a) Architecture of microwave-speech transformer (MST) inspired by Ref.[1]. **b)** Architecture of the CNN used the recognize the speaker's identity from the acquired microwave biosignals. **c**) Architecture of the CNN used to reconstruct the skeleton of the subject from the microwave biosignals. BN denotes batch normalization, $k(a, b, c)$ denotes a convolutional kernel of size $a \times b \times c$, and $n(a)$ denotes the number of convolutional kernels as a, and CNN-t denotes the transpose operation of the CNN.

●The CNN to recognize the speaker's identity

Fig. S1b shows the architecture of the CNN that we use to recognize the speaker's identification based on the acquired microwave biosignals. The CNN maps the sequence of microwave biosignals to the label of the speaker of interest in an end-to-end fashion. The CNN consists of three convolution layers (yellow), three pooling layers (blue), a full connection layer (red), and a Softmax layer (cyan). The size of original microwave data is 201×100×2, in which 201 is the number of frequency sampling points, 100 is the number of time sampling points, and 2 refers to the real and imaginary parts of the complex-valued microwave biodata. After being processed by three convolutional layers, the size of data is 23×10×16, with 3680 parameters in total. Then, this data is injected into a fully connected layer with size 3680×128 to obtain a vector of length 128. Finally, we utilize a Softmax layer with size 128×7 to get a vector of length 7.

●The CNN for the reconstruction of the speaker's 3D skeleton

Fig. S1c shows the architecture of the CNN used for reconstructing the speaker's skeleton. The speaker is illuminated with 18 random wavefronts generated by a fixed sequence of 18 random metasurface configurations. The CNN shown in **Fig. S1c** directly maps this microwave data to the speaker's 3D skeleton in an end-to-end fashion. To obtain labeled training data, a binocular camera (ZED2 by StereoLabs) was used in the experiment to collect the 3D skeleton information of the human body. The 3D skeleton contains 18 key points of the human body: nose, neck, right shoulder, right elbow, right wrist, left shoulder, left elbow, left wrist, right hip, right knee, right ankle, left hip, left knee, left ankle, right eye, left eye, right ear, left ear. We collected 20,000 training samples in our lab environment to train the network, including scenarios with one and two speaker(s). The tests of our microwave-based 3D skeleton reconstruction went well and manage to track each key skeleton point on the human body with an average error of 4 cm.

Supplementary Note 2: Design of meta-atom and programmable metasurface

In this supplementary note, we elaborate on our electronically-controllable binary-phase meta-atom in terms of its design parameters and fabrication. As shown in **Fig. S2a**, the designed meta-atom consists of a five-layer structure. The top square copper patch with dimensions of 11×11 mm^2 is integrated with a MADP-000907-14020x diode connected to the ground plane via a hole. The PIN diode can be modelled as a series lumped-parameter circuit. When the diode is switched ON, it is represented by a 30pH inductor in series with a 7.8 Ω resistor. In contrast, when the PIN diode is switched at OFF, it is modeled by a 28pF capacitor in series with a 30pH inductor. The Layer-2 with thickness of 1.58 mm is Taconic TLX-8 with a relative permittivity of 2.55. The Layer-4 with thickness of 0.3 mm is FR-4 with a dielectric constant of 4.3. The Layer-3 and Layer-5 are ground planes mode of copper, and a via hole is introduced on the Layer-3 to isolate the bias voltage coming from Layer-5. As demonstrated in **Fig. 1** in main text, we observe that the reflection phase of the designed meta-atom will experience a 180° phase difference when the PIN diode is switched from ON (OFF) to OFF (ON) in the frequency range between 7.49 GHz and 8.3 GHz. The phase change can be accomplished by switching the external DC voltage applied to the PIN diode from 5 V to 0 V.

As shown in **Fig. S2b**, the whole programmable metasurface consists of 32×32 independently electronically-controllable meta-atoms. Since each meta-atom has a size of 16×16 mm^2, the whole metasurface has size of 512×512 mm^2 in total. We remark that the whole programmable metasurface is composed of 2×2 identical panels due to fabrication restrictions, and each panel has 16×16 meta-atoms. The whole programmable metasurface is electronically controlled with an FPGA-based Micro-Control-Unit (MCU). An FPGA chip is used to distribute all commands to the 1024 PIN diodes. To achieve real-time and flexible controls of 1024 PIN diodes soldered onto the programmable metasurface, the MCU with size of 90×90 mm^2 is designed and assembled on the upper rear of the metasurface. The MCU is responsible for dispatching all commands sent from a master computer subject to one common clock (CLK) signal. In our work, the adopted CLK is 50 MHz, and the switching time of a PIN diode is about 10 μs in each cycle.

Each metasurface panel is equipped with eight 8-bit shift registers (SN74LV595APW), as shown in **Fig. S2c**, and every 32 PIN diodes are divided into 8 groups sharing the same shift register. With the use of shift registers, 8 groups of PIN diodes are controlled in a sequential manner. Then the MCU will send the commands over 16 independent branch channels, leading to the almost real-time manipulation of all PIN diodes. In addition, 1024 red-color LEDs are soldered onto the metasurface to indicate the status of the associated PIN diodes, revealing clearly whether the PIN diode works well or not.

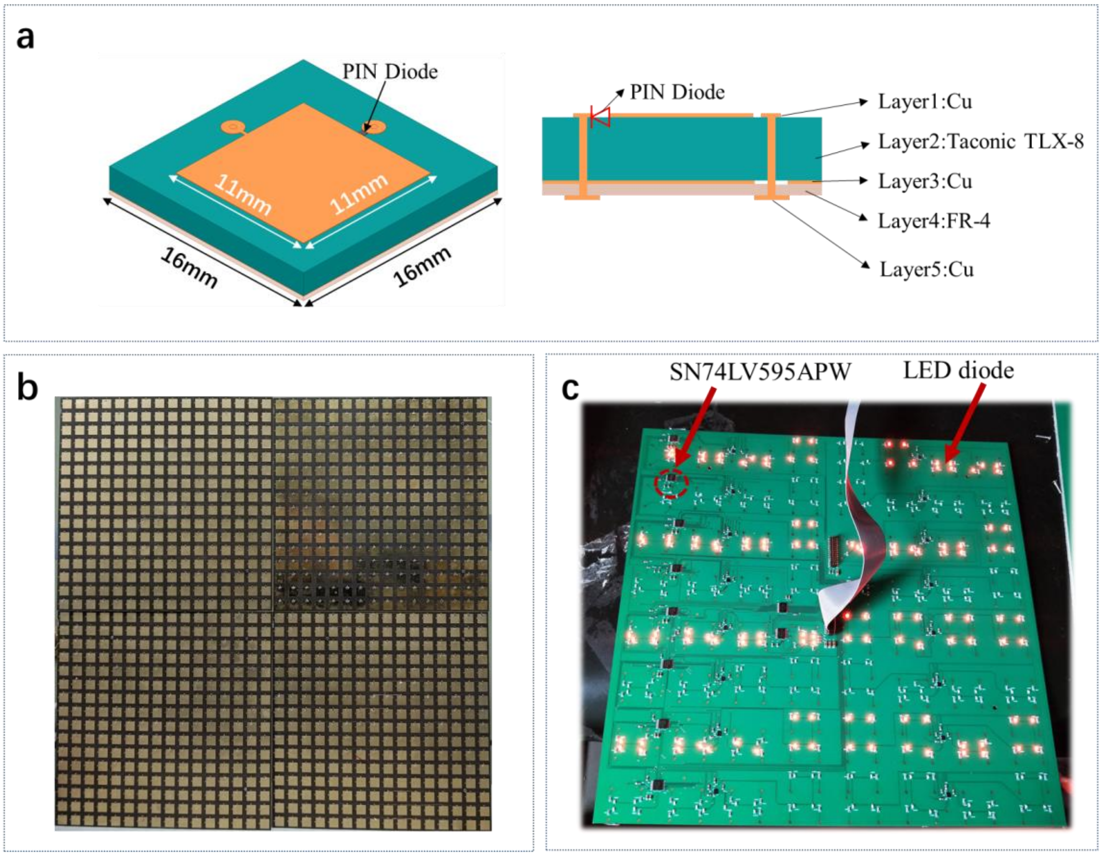

Fig. S2. Design of meta-atom and programmable metasurface. **a)** Schematic drawing of the designed one-bit programmable meta-atom. **b)** Photographic image of the front side of the designed programmable metasurface. **c)** Photographic image of the back side of one metasurface panel.

Supplementary Note 3: Designated material of 100 daily used English words used in our microwave speech recognition experiment

The designated material is a list of 100 daily used English words shown in **Supplementary Table 1**. All of the experiments in our work is based on this material. We think that, since the recognition accuracy of our system is rather high, these 100 words are definitely not the limitation of the system. In the future work, we may build a larger dataset and do a more detailed research on physical mechanism of microwave lip reading based on programmable metasurface.

Table S1. The list of 100 daily English words used as the designated material.

accurate /ˈækjərət/	dust /dʌst/	image /ˈɪmɪdʒ/	rocket /ˈrɒkɪt/
acquire /əˈkwaɪə(r)/	effective /ɪˈfektɪv/	import /ˈɪmpɔːt/	running /ˈrʌnɪŋ/
angle /ˈæŋgl/	enlarge /ɪnˈlɑːrdʒ/	inverse /ˌɪnˈvɜːs/	screen /skriːn/
arrange /əˈreɪndʒ/	equality /iˈkwɒlətɪ/	january /ˈdʒænjʊərɪ/	significant /sɪgˈnɪfɪkənt/
attension /əˈtenʃn/	especially /ɪˈspeʃəlɪ/	juice /dʒuːs/	source /sɔːs/
balance /ˈbæləns/	export /ɪkˈspɔːt/	keyboard /ˈkiːbɔːd/	subject /ˈsʌbdʒɪkt/
before /bɪˈfɔːr/	face /feɪs/	kind /kaɪnd/	temperature /ˈtemprətʃə(r)/
better /ˈbetə(r)/	feather /ˈfeðə(r)/	latitude /ˈlætɪtjuːd/	theatre /ˈθɪətə(r)/
bright /braɪt/	february /ˈfebrʊərɪ/	level /ˈlevl/	thesis /ˈθiːsɪs/
button /ˈbʌtn/	foster /ˈfɒstə(r)/	look /lʊk/	think /θɪŋk/
canvas /ˈkænvəs/	fridge /frɪdʒ/	magic /ˈmædʒɪk/	transform /trænsˈfɔːm/
channel /ˈtʃænl/	full /fʊl/	maximum /ˈmæksɪməm/	treasure /ˈtreʒə(r)/
classical /ˈklæsɪkl/	gain /geɪn/	multiply /ˈmʌltɪplaɪ/	tube /tjuːb/
cloud /klaʊd/	generalize /ˈdʒenrəlaɪz/	natural /ˈnætʃrəl/	turn /tɜːn/
compare /kəmˈpeə(r)/	gesture /ˈdʒestʃə(r)/	neighborhood /ˈneɪbəhʊd/	update /ˌʌpˈdeɪt/
compute /kəmˈpjuːt/	gift /gɪft/	noise /nɔɪz/	useful /ˈjuːsfl/
content/ˈkɒntent/	glass /glɑːs/	number /ˈnʌmbə(r)/	usually /ˈjuːʒʊəlɪ/
contrast /ˈkɒntrɑːst/	golden /ˈgəʊldən/	oasis /əʊˈeɪsɪs/	various /ˈveərɪəs/
dark /dɑːk/	graph /grɑːf/	operate /ˈɒpəreɪt/	vertical /ˈvɜːtɪkl/
define /dɪˈfaɪn/	grow /grəʊ/	pair /peə(r)/	visual /ˈvɪʒuəl/

different /ˈdɪfrənt/	guidance /ˈgaɪdns/	parameter /pəˈræmɪtə(r)/	wake /weɪk/
displace /dɪsˈpleɪs/	head /hed/	performance /pəˈfɔːməns/	water /ˈwɔːtə(r)/
document /ˈdɒkjumənt/	hospital /ˈhɒspɪtl/	purpose /ˈpɜːpəs/	willing /ˈwɪlɪŋ/
drop /drɒp/	human /ˈhjuːmən/	radio /ˈreɪdiəʊ/	zero /ˈzɪərəʊ/
duplicate /ˈduːplɪkeɪt/	identity /aɪˈdentəti/	risk /rɪsk/	zoom /zuːm/

Supplementary Note 4: Some microwave-audio samples

In this supplementary note, some selected pairs of microwave-audio signals are presented in **Fig. S3**, which were collected by using our metasurface-empowered microwave speech recognizer. Here, we randomly chose four people (2 females and 2 males) from 22 volunteers, and we provide their voice and microwave signals for four typical sentences from more than 100 words. Each sample's duration is about 10s. We plot amplitude (top) and phase (middle) of the microwave biosignal as well as the corresponding audio signal (bottom).

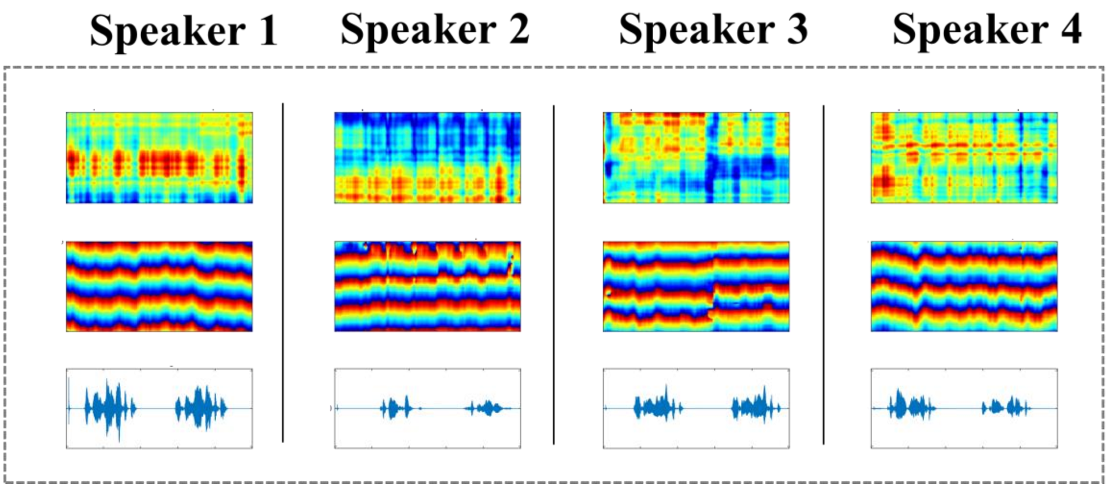

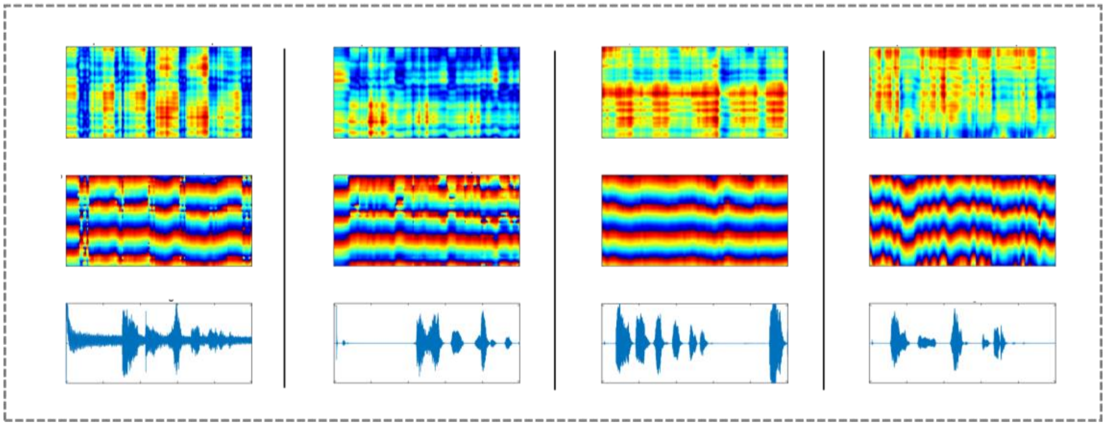

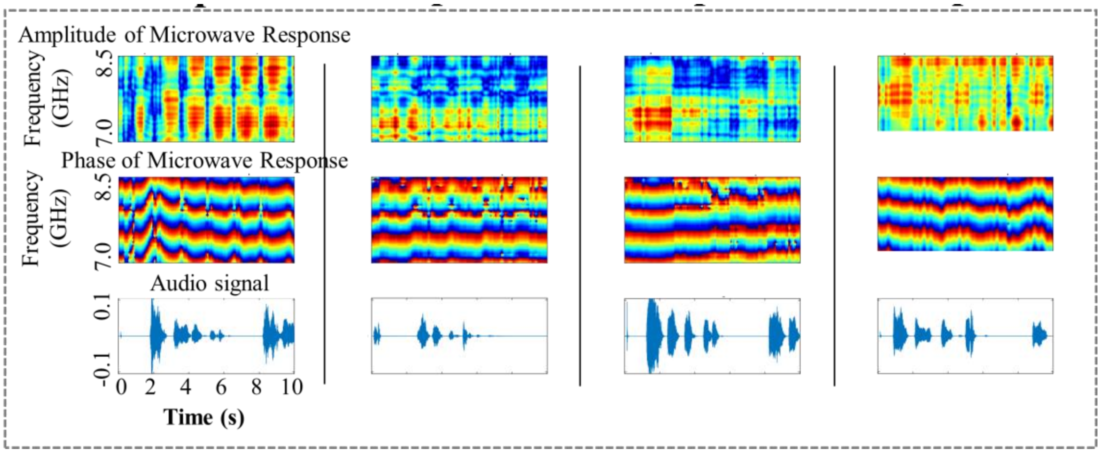

Fig. S3. Selected pairs of microwave-audio samples. For four speakers and three text samples, we plot amplitude (top) and phase (middle) of the acquired microwave biosignal, as well as the corresponding sound signal (bottom) acquired by the in-built microphone of our host computer.

Supplementary Note 5: Speech sensing results in through-wall setting

In this supplementary note, we consider the microwave speech recognition in a through-wall setting where two subjects sit behind a 5cm-thick wooden wall. In this setting, the spatial energy of microwave signal from the transmitter must be simultaneously focused on the mouths of both subjects by properly manipulating the coding pattern of the one-bit reprogrammable metasurface. A set of experimental results for which the mouths of the two speakers are located at around (-5 cm, 16.2 cm, 65 cm) and (15 cm, 16.2 cm, 65 cm), respectively, is shown in **Fig. 1e**.

To train our metasurface-empowered microwave speech recognizer, 22 participants were invited to read the assigned English reading material five times behind a 5cm-thick wooden

wall. Similar to the previous processing, 70% of the samples are randomly selected for training the microwave-voice transformer, and the rest are used for testing. The corresponding experimental results have been reported in **Fig. S4**. **Fig. S4** plots the performance of the training (dotted line) and generalization (dashed line) of the developed microwave speech recognition system in terms of the value of the loss function as the index of the training epoch increases. The dependence of the recognition accuracy over the testing samples on the index of the training epoch has been plotted as well. We can see that the speech information of the subject behind a wall can be recognized with an accuracy of above 80 % by the developed microwave speech recognizer. In addition, to examine the effect on the accuracy of the speech recognition from the clutters of the ambient environment, a set of experiments has been conducted where a third person acts freely in the room while the two subjects talk to each other. The corresponding results are plotted in **Fig. S4** as well. These results yield the important conclusion that the developed microwave speech recognizer is robust to clutter and noise induced through a dynamic environment. This robustness can be attributed to our use of a large-aperture reprogrammable metasurface along with a beamforming technique that is very efficient at focusing the microwaves on the speakers' mouths.

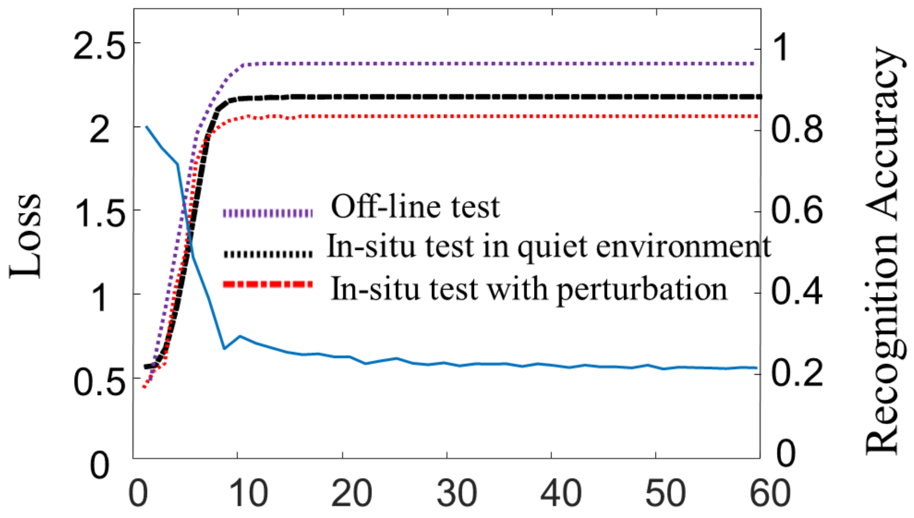

Fig. S4. Training and testing behaviors of our microwave speech recognizer as a function of the training epoch. The loss function is plotted as blue solid line (left axis), and the test behavior is examined in terms of the recognition accuracy (right axis). We test our microwave speech recognizer trained in the quiet

environment in three scenarios: first, the simple test with the off-line collected test samples (called off-line test); second, the in-situ test with a subject in the same quiet environment as that for the training (called in-situ test in quiet environment); third, the in-situ test with a subject disturbed by an additional person freely acting in room (called in-situ test with perturbation).

Supplementary Note 6: Human-robot interaction

In this supplementary note, we elaborate on the application of the developed metasurface-empowered microwave speech recognizer to human-robot interaction. As shown in **Fig. S5a**, a bionic mechanical hand is integrated into a mobile vehicle and each finger is fitted with an anti-blocking joint servo (LFD-01) for finger retraction control. An on-board WiFi module (nRF24L01) is utilized to receive the control commands from the host computer and feedback the state of the mechanical hand. The vehicle is equipped with an STM32 controller to process the received control commands into control quantities for the corresponding servos. We experimentally examine the performance of real-time human-robot interaction using our microwave speech metasurface recognizer. The signal flow diagram of the individual modules of the mechanical hand is shown in **Fig. S5b**. The test subject sequentially utters five different speech commands: ‘one’, ‘two’, ‘three’, ‘four’, and ‘five’. The speech commands are recognized by our metasurface-empowered microwave speech recognizer and then transmitted in real time to the mobile vehicle by the WiFi wireless communication module carried on the host computer; the vehicle carries the same WiFi module to receive the commands. The commands are then input to the STM32 controller and we group the control commands of the joint servos corresponding to the specific gestures of the mechanical hand, enabling the control of the corresponding gestures according to the different speech commands via the STM32 controller. Finally, the mechanical hand feeds the completed gesture status back to the host computer. Corresponding experimental results are presented in **Fig. S5c**, and more results can be found in **Supplementary Video 1**.

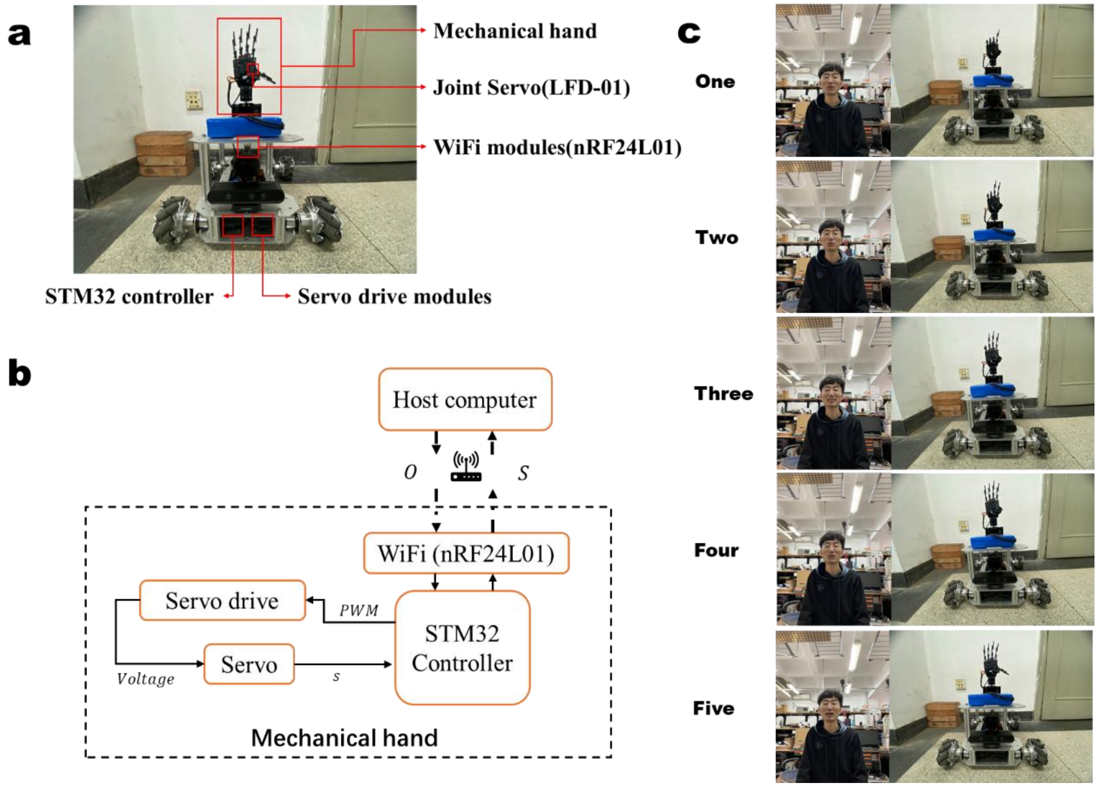

Fig. S5. a) Mobile vehicle experimental platform with a mechanical hand. **b**) Control flow diagram of the mechanical hand. **c**) Results of real-time interaction between a human and a robotic mechanical hand based on human speech commands captured by our metasurface-empowered microwave speech recognizer. Here, PWM: pulse waveform modulator, o/O: observation; s/S: state.

Supplementary Note 7: Experimental results on privacy-preserving properties of microwave speech recognition

The deep neural network developed in this work has really good generalizability. In this way, the lip language recognition process doesn't need the enrollment of the subject, which means that lip language collected from a person unrecorded by our system can still be recognized with high accuracy. In particular, the training data consists of data from 22 participants, but for an unknown person, the model is able to work with rather high accuracy as well when the unknown subject speaks the 100 daily-used English words. To see it clearly, we have designed this experiment to see if the microwave lip language from an unknown person can be accurately recognized. The recognition result is shown in **Fig. S6**. Here, we still use the aforementioned 22 participants, but take randomly 14 participants for training, and the rest of 8 participants for testing. The data from these 8 testing

participants are organized into 4 groups. Each group contains all data of 2 participants. The recognition accuracy of the four groups of test samples is about 90% when wearing masks or not. The result indicates that regardless of whether participants wear masks or not, the model is able to recognize random subjects' lip language accurately. Therefore, we can say that our system can be regarded as universal.

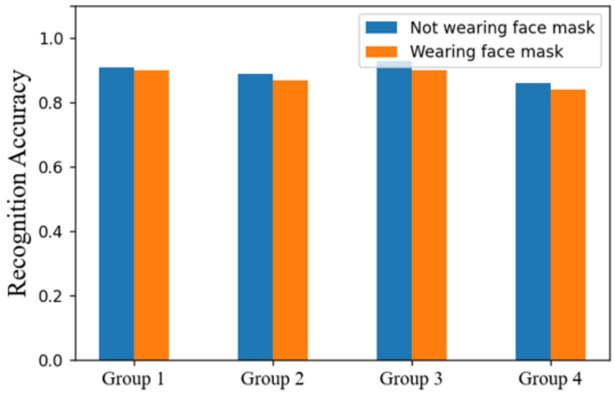

Fig. S6. Experimental results of the pretrained microwave-speech transformer when working for unseen subjects.

Supplementary References

1. Vaswani, A. *et al.* Attention is All you Need. *Proc. NIPS* 11 (2017).
